# Supplementary material for: Uncovering the socioeconomic facets of human mobility
Source: Sci Rep. 2021 Apr 21;11:8616. doi: 10.1038/s41598-021-87407-4 (PMC8060260; doi:10.1038/s41598-021-87407-4)
Supplement: Supplementary file 1 — Supplementary Information. [file 41598_2021_87407_MOESM_ESM.pdf]

Supporting Information

Uncovering the socioeconomic facets of human mobility

Hugo Barbosa, Surendra Hazarie, Brian Dickinson, Aleix Bassolas, Adam Frank, Henry Kautz, Adam Sadilek, José J. Ramasco, and Gourab Ghoshal

S1 Census data and income metrics

To represent socioeconomic status we collected information on median income, age, sex composition and race from the 2016 5-year American Community Survey (ACS) estimates for the United States and the 2010 decennial census for Brazil [38,39]. The full set of cities are shown in Tabs. S1 and S2. An example of the data for the city of Rochester, NY is shown in Tab. S3, and for Campinas, Brazil in Tab. S4. The spearman correlation between these variables indicates a strong correlation with median income in both the United States (Fig. S1) and Brazil (Fig. S2).

To capture income, we utilize two different representations of the income data, both obtained from the nominal income household variables. The first is the fraction f_i of the populations within an area having a per capita income within one of the n income brackets, as defined by the corresponding census authorities, such that $\sum_{i=1}^{n} f_1 = 1$. For the case of the US, there were $n = 8$ income breaks, ranging from below 10k to above 75kUSD per year. Therefore, each area in the data is characterized by a vector of n values. For instance, if in a given area in the US data (e.g., ZCTA) has an income breakdown vector

$$I = [0.09, 0.1, 0.15, 0.15, 0.25, 0.15, 0.10, 0.01],$$

it means that 9 % of that population has a income per-capita below 10k USD a year whereas 1 % has an income above 75k USD. We refer to this fraction-based income variable as ID_f.

Similarly for Brazil, household-level incomes are partitioned into $n = 9$ intervals relative to the Brazilian National Minimum Wage (NMW), ranging from less than 1/2 NMW to nominal incomes above 20 NMWs. The second variable is extracted from the median income per capita of an area (ZCTA or WA). From this median income, we split the regions into five income quantiles, which we label as *low* (bottom 20%), *mid-low* (between 20%-40%), *mid* (between 40%-60%), *mid-high* (between 60%-80%) and *high* (above 80%). These income quantiles are relative to each individual city, accounting therefore for the socioeconomic differences across cities. This metric, referred to as ID_q allows us to label an area of a city according to its overall income characteristics.

	Urban Area	Population
1	Albany–Schenectady, NY Urbanized Area	669473
2	Allentown, PA–NJ Urbanized Area	822617
3	Atlanta, GA Urbanized Area	5381110
4	Austin, TX Urbanized Area	1711701
5	Baltimore, MD Urbanized Area	2294493
6	Birmingham, AL Urbanized Area	855126
7	Boston, MA–NH–RI Urbanized Area	4704737
8	Bridgeport–Stamford, CT–NY Urbanized Area	1019263
9	Buffalo, NY Urbanized Area	1038718
10	Charlotte, NC–SC Urbanized Area	1747070
11	Chicago, IL–IN Urbanized Area	8917324
12	Cincinnati, OH–KY–IN Urbanized Area	1848639
13	Cleveland, OH Urbanized Area	1999159
14	Columbus, OH Urbanized Area	1512446
15	Dallas–Fort Worth–Arlington, TX Urbanized Area	5996274
16	Denver–Aurora, CO Urbanized Area	2681764
17	Detroit, MI Urbanized Area	4035863
18	Hartford, CT Urbanized Area	1105969
19	Houston, TX Urbanized Area	5412762
20	Indianapolis, IN Urbanized Area	1690182
21	Kansas City, MO–KS Urbanized Area	1645871
22	Las Vegas–Henderson, NV Urbanized Area	1905136
23	Los Angeles–Long Beach–Anaheim, CA Urbanized...	12686477
24	Louisville/Jefferson County, KY–IN Urbanized ...	1078499
25	Miami, FL Urbanized Area	5832060
26	Milwaukee, WI Urbanized Area	1486454
27	Minneapolis–St. Paul, MN–WI Urbanized Area	2933169
28	New Orleans, LA Urbanized Area	967547
29	New York–Newark, NY–NJ–CT Urbanized Area	18987636
30	Oklahoma City, OK Urbanized Area	1125647
31	Omaha, NE–IA Urbanized Area	803087
32	Orlando, FL Urbanized Area	1866002
33	Philadelphia, PA–NJ–DE–MD Urbanized Area	5821389
34	Phoenix–Mesa, AZ Urbanized Area	4038319
35	Pittsburgh, PA Urbanized Area	1955719
36	Portland, OR–WA Urbanized Area	2080902
37	Providence, RI–MA Urbanized Area	1188589
38	Rochester, NY Urbanized Area	806269
39	Sacramento, CA Urbanized Area	1889988
40	San Antonio, TX Urbanized Area	2091059
41	San Diego, CA Urbanized Area	3097903
42	San Francisco–Oakland, CA Urbanized Area	3508790
43	San Jose, CA Urbanized Area	1733914
44	Seattle, WA Urbanized Area	3429707
45	Springfield, MA–CT Urbanized Area	674903
46	St. Louis, MO–IL Urbanized Area	2309620
47	Tampa–St. Petersburg, FL Urbanized Area	2689944
48	Virginia Beach, VA Urbanized Area	1554524
49	Washington, DC–VA–MD Urbanized Area	5098266
50	Worcester, MA–CT Urbanized Area	541879

Table S1: List of US cities and their populations

	Urban Area	Population
1	Aglomerao Urbana de So Lus	1014741
2	Anpolis	332727
3	Bauru	343877
4	Belm	1865652
5	Belo Horizonte	3854722
6	Blumenau	308402
7	Bragana Paulista	160665
8	Braslia	2566723
9	Campinas	1924733
10	Campo Grande	785683
11	Caraguatatuba	113317
12	Caxias do Sul	435791
13	Cuiab	803497
14	Curitiba	2227447
15	Florianpolis	629936
16	Fortaleza	2985515
17	Goinia	1757432
18	Guarulhos	1222308
19	Ilhus-Itabuna	204310
20	Itaja	5544
21	Itapecerica da Serra	714128
22	Joinville	515128
23	Joo Pessoa	722866
24	Juiz de Fora	516417
25	Jundia	369862
26	Lagos	14368000
27	Londrina	507114
28	Macei	931636
29	Manaus	1793000
30	Mogi das Cruzes	968510
31	Natal	1006137
32	Osasco	1474647
33	Osrio	40000
34	Piracicaba	363890
35	Porto Alegre	2876717
36	Presidente Prudente	207841
37	Recife	2808854
38	Ribeiro Preto	604494
39	Rio de Janeiro	10816739
40	Salvador	2917528
41	Santos	1297281
42	So Jos do Rio Preto	408079
43	So Jos dos Campos	1118953
44	So Paulo	12698814
45	Sorocaba	586084
46	Teresina	812594
47	Tubaro	104937
48	Uberlndia	604148
49	Vale do Paraba Fluminense	232296
50	Vitria	1497160

Table S2: List of BR cities and their populations

	Urban Area	ZCTA	Median Income ($)	Fraction Bachelors degree	Median Age	Fraction White
8429	Rochester, NY	14424	29692.0	0.177	46.3	0.948
8430	Rochester, NY	14425	34762.0	0.264	37.0	0.913
8431	Rochester, NY	14428	32452.0	0.209	47.1	0.977
8432	Rochester, NY	14445	31355.0	0.196	35.9	0.883
8433	Rochester, NY	14450	39894.0	0.302	45.3	0.930
8434	Rochester, NY	14467	32915.0	0.189	39.8	0.770
8435	Rochester, NY	14468	32431.0	0.163	42.7	0.970
8436	Rochester, NY	14502	33661.0	0.197	41.2	0.963
8437	Rochester, NY	14506	59107.0	0.355	45.9	0.986
8438	Rochester, NY	14514	33598.0	0.208	41.3	0.890
8439	Rochester, NY	14519	30990.0	0.160	44.6	0.948
8440	Rochester, NY	14526	40069.0	0.258	44.5	0.919
8441	Rochester, NY	14534	47629.0	0.328	46.1	0.888
8442	Rochester, NY	14543	36729.0	0.242	50.4	0.965
8443	Rochester, NY	14544	28083.0	0.102	43.1	0.988
8444	Rochester, NY	14546	31126.0	0.191	41.8	0.874
8445	Rochester, NY	14548	30110.0	0.114	48.3	0.973
8446	Rochester, NY	14559	34586.0	0.229	42.9	0.954
8447	Rochester, NY	14564	40391.0	0.270	42.5	0.954
8448	Rochester, NY	14568	32311.0	0.244	37.9	0.983
8449	Rochester, NY	14580	35109.0	0.250	43.5	0.913
8450	Rochester, NY	14586	36719.0	0.255	32.9	0.756
8451	Rochester, NY	14589	27301.0	0.124	45.3	0.928
8452	Rochester, NY	14604	16330.0	0.083	43.9	0.550
8453	Rochester, NY	14605	11290.0	0.073	25.8	0.285
8454	Rochester, NY	14606	23630.0	0.109	36.4	0.631
8455	Rochester, NY	14607	27811.0	0.320	30.0	0.799
8456	Rochester, NY	14608	15074.0	0.128	29.6	0.225
8457	Rochester, NY	14609	25608.0	0.146	33.5	0.544
8458	Rochester, NY	14610	39575.0	0.266	44.1	0.875
8459	Rochester, NY	14611	16054.0	0.062	30.7	0.267
8460	Rochester, NY	14612	31247.0	0.201	45.5	0.894
8461	Rochester, NY	14613	19657.0	0.097	30.5	0.400
8462	Rochester, NY	14614	8894.0	0.041	28.7	0.360
8463	Rochester, NY	14615	23572.0	0.148	35.9	0.634
8464	Rochester, NY	14616	28127.0	0.159	41.5	0.847
8465	Rochester, NY	14617	34438.0	0.272	44.2	0.930
8466	Rochester, NY	14618	36906.0	0.274	40.1	0.859
8467	Rochester, NY	14619	22250.0	0.114	32.6	0.199
8468	Rochester, NY	14620	24646.0	0.252	31.4	0.714
8469	Rochester, NY	14621	15884.0	0.060	33.0	0.343
8470	Rochester, NY	14622	30134.0	0.176	46.4	0.881
8471	Rochester, NY	14623	21566.0	0.215	27.7	0.722
8472	Rochester, NY	14624	30507.0	0.204	41.2	0.838
8473	Rochester, NY	14625	36157.0	0.272	48.4	0.956
8474	Rochester, NY	14626	30845.0	0.176	47.0	0.886

Table S3: Socioeconomic variables for zip codes in Rochester, NY

	Urban Area	Area Code	Median Income ($)	Fraction Bachelors degree	Median Age	Fraction White
1287	Campinas	3501608005001	1500.0	0.278	41.0	0.913
1288	Campinas	3501608005002	1000.0	0.255	33.0	0.714
1289	Campinas	3501608005003	850.0	0.209	30.0	0.698
1290	Campinas	3501608005004	1200.0	0.262	33.0	0.791
1291	Campinas	3501608005005	1500.0	0.270	33.0	0.795
1292	Campinas	3501608005006	1000.0	0.249	31.0	0.733
1293	Campinas	3501608005007	1400.0	0.231	36.0	0.815
1294	Campinas	3501608005008	1500.0	0.295	38.0	0.907
1295	Campinas	3501608005009	1500.0	0.290	38.0	0.862
1296	Campinas	3501608005010	1000.0	0.252	32.0	0.743
1342	Campinas	3509502005001	940.0	0.173	33.0	0.657
1343	Campinas	3509502005002	1500.0	0.249	36.0	0.802
1344	Campinas	3509502005003	1750.0	0.258	32.0	0.770
1345	Campinas	3509502005004	2500.0	0.221	34.0	0.812
1346	Campinas	3509502005005	2000.0	0.266	39.0	0.782
1347	Campinas	3509502005006	2000.0	0.259	34.0	0.804
1348	Campinas	3509502005007	1850.0	0.283	37.0	0.860
1349	Campinas	3509502005008	1200.0	0.228	34.0	0.743
1350	Campinas	3509502005009	1500.0	0.290	36.0	0.787
1351	Campinas	3509502005010	2500.0	0.261	37.0	0.874
1352	Campinas	3509502005011	2850.0	0.187	35.0	0.836
1353	Campinas	3509502005012	4200.0	0.216	42.0	0.906
1354	Campinas	3509502005013	1700.0	0.325	36.0	0.799
1355	Campinas	3509502005014	2000.0	0.281	39.0	0.897
1356	Campinas	3509502005015	1110.0	0.263	32.0	0.716
1357	Campinas	3509502005016	1200.0	0.291	37.0	0.747
1358	Campinas	3509502005017	1000.0	0.245	31.0	0.622
1359	Campinas	3509502005018	1000.0	0.262	31.0	0.641
1360	Campinas	3509502005019	1500.0	0.269	33.0	0.712
1361	Campinas	3509502005020	1510.0	0.310	37.0	0.793
1362	Campinas	3509502005021	1000.0	0.247	31.0	0.651
1363	Campinas	3509502005022	1500.0	0.272	35.0	0.787
1364	Campinas	3509502005023	1200.0	0.270	34.0	0.724
1365	Campinas	3509502005024	800.0	0.170	26.0	0.457
1366	Campinas	3509502005025	900.0	0.239	28.0	0.499
1367	Campinas	3509502005026	826.4	0.192	27.0	0.499
1368	Campinas	3509502005027	900.0	0.200	27.0	0.470
1369	Campinas	3509502005028	1000.0	0.273	30.0	0.512
1370	Campinas	3509502005029	860.0	0.192	27.0	0.484
1371	Campinas	3509502005030	900.0	0.205	28.0	0.484
1372	Campinas	3509502005031	850.0	0.208	29.0	0.512
1373	Campinas	3509502005032	700.0	0.134	24.0	0.378
1374	Campinas	3509502005033	930.0	0.226	28.0	0.585
1375	Campinas	3509502005034	1000.0	0.229	31.0	0.572
1376	Campinas	3509502005035	1100.0	0.271	31.0	0.659
1377	Campinas	3509502005036	800.0	0.195	27.0	0.458
1495	Campinas	3519071005001	1200.0	0.296	31.0	0.680
1496	Campinas	3519071005002	1000.0	0.280	30.0	0.549
1497	Campinas	3519071005003	1000.0	0.272	30.0	0.608
1498	Campinas	3519071005004	839.0	0.191	27.0	0.475

Table S4: Socioeconomic variables for area codes in Campinas

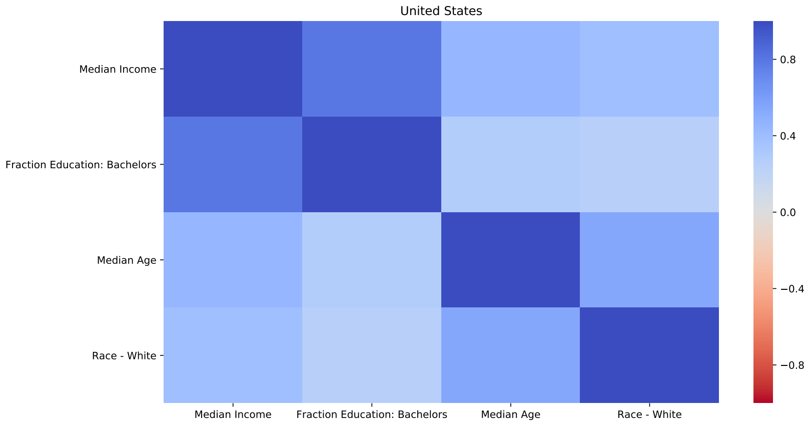

Figure S1: Spearman correlations between 4 selected dimensions of the survey data taken from 50 American cities.

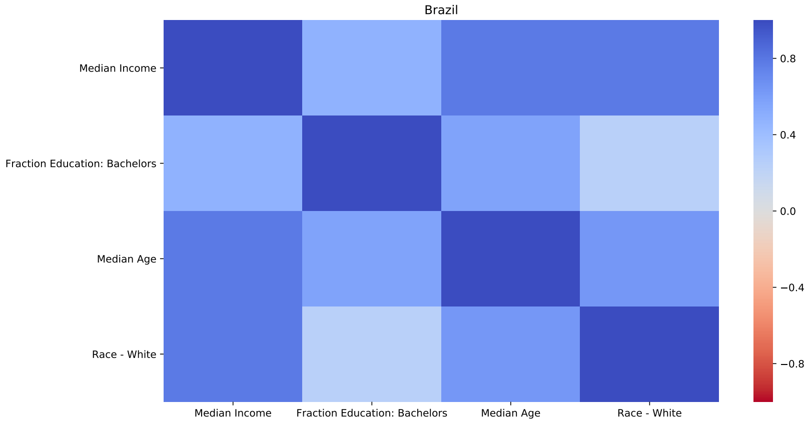

Figure S2: Spearman correlations between 4 selected dimensions of the survey data taken from 50 Brazilian cities.

S2 Basic amenities data

	Atlanta, GA	Boston, MA	Detroit, MI	Rochester, NY
marketplace	15	13	9	0
restaurant	1020	1447	566	182
food court	2	6	0	0
fast food	534	513	341	64
hospital	6	13	7	0
doctors	39	49	77	6
dentist	23	67	20	4
clinic	21	18	40	3
pharmacy	107	129	96	16
kindergarten	11	18	4	2
school	1231	1253	1253	339
college	11	22	3	5
university	4	18	3	1
library	84	648	41	36
bank	124	212	103	44
atm	234	164	36	27

Table S5: Number of basic amenities by category for Atlanta, GA, Boston MA, Detroit, MI, and Rochester, NY.

S3 Connecting outflow and resident population based on income

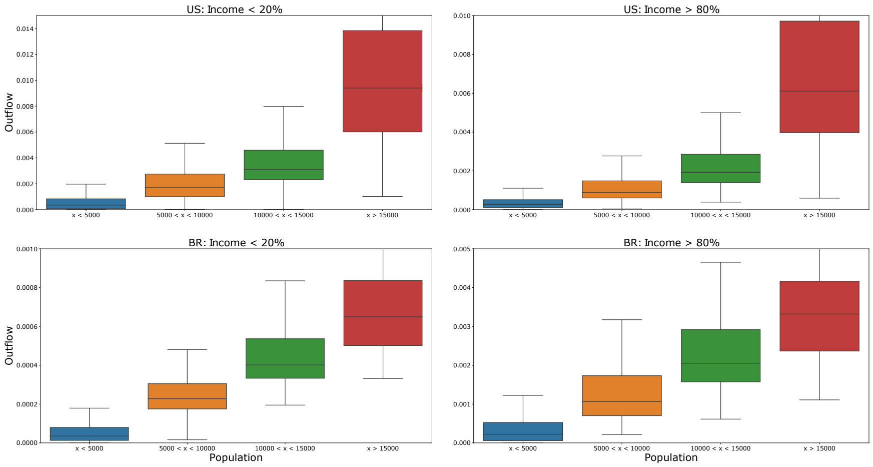

Figure S3: Box-plots of mobility outflows for the top and bottom 20% of zip codes in terms of median income, for the US (top) and Brazil (bottom.) The data is binned by population, and the boxes indicate quartiles of the distributions. We see that in all four cases, population is positively correlated with mobility (Pearson correlations: US $< 20\% : .85, > 80\% : .73$; BR $< 20\% : .81, > 80\% : .67$) indicating that mobility is representative of population regardless of income.

S4 Cluster analysis

The clustering of cities was done using a divisive hierarchical method to split the cities into disjoint groups of urban areas with similar patterns in their mobility/socioeconomic correlations. To determine the ideal number of partitions we use a silhouette analysis. The silhouette value is a measure of how similar a data point is to the cluster it has been assigned to in comparison to its average distance to another candidate cluster. More precisely, the silhouette score $s(i)$ of a data point i with regards do a partitioning structure C can be defined as

$$s(i) = \frac{b(i) - a(i)}{\max(a(i), b(i))}$$

where

$$a(i) = \frac{1}{|C_i| - 1} \sum_{j \in C_i, i \neq j} d(i, j)$$

is the mean distance $d(i, j)$ between the point i and all other points (j) in the same cluster C_i and

$$b(i) = \min_{k \neq i} \frac{1}{|C_k|} \sum_{j \in C_k} d(i, j)$$

where $b(i)$ is the minimum average distance of the point i to the points of one of the other clusters. Here, $b(i)$ is the distance of i to the cluster it is the most similar with, other than the one it has been assigned to. Therefore, $s(i) \in [-1, 1]$ such that if the silhouette score $s(i) > 0$ that data point is in the best cluster it could belong to. Conversely, if $s(i) < 0$ the point i is not assigned to its most natural cluster, suggesting that the partitioning structure is not ideal. The ideal partitioning structure is expected to be the one that produces only positive silhouette scores at the same time that maximizes the average silhouette score. Figure S4 shows the average silhouette scores for $k = 2$ to $k = 10$. For both countries $k = 2$ produces the maximum average silhouette score.

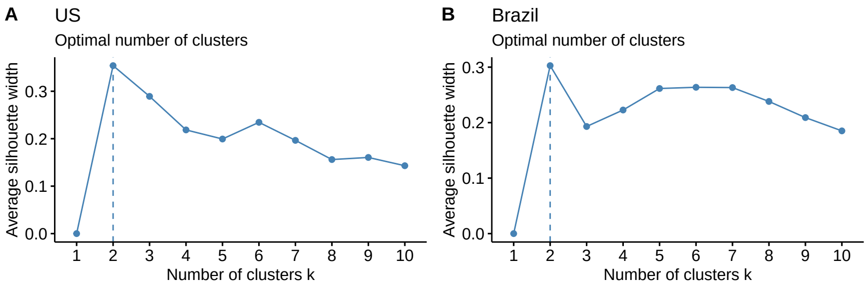

Figure S4: Average Silhouette scores for the partitioning structures produced by the divisive hierarchical clustering algorithm as a function of the number of clusters, k For both the US **(A)** and Brazil **(B)** the best partition is obtained for $k = 2$.

S5 Commuting data

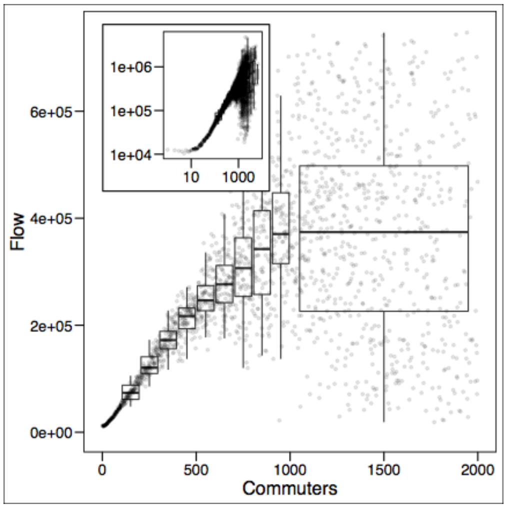

Figure S5: (The mobility flows from the location based data as a function of the commuting flow extracted from the United States census.

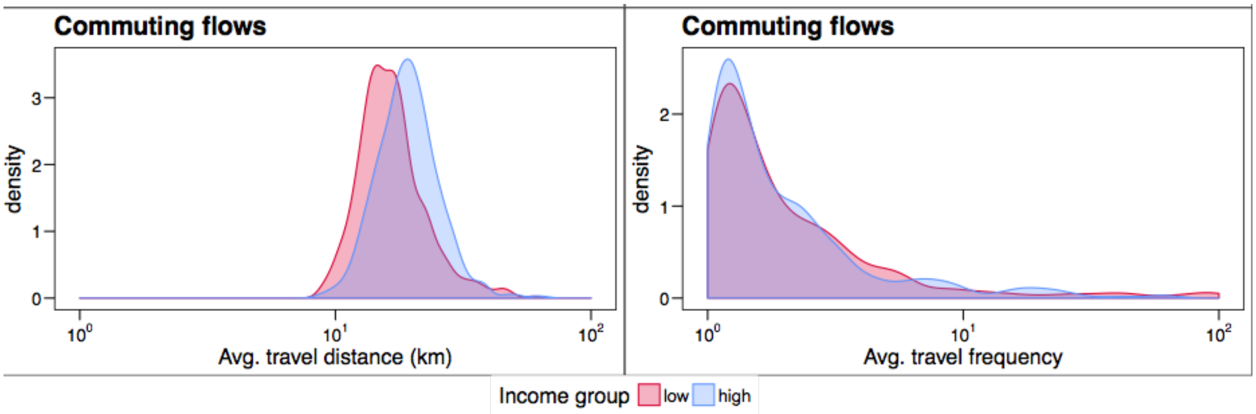

Figure S6: Average travel distance across income groups matches the trends seen in Fig. 2, where higher income groups travel longer distances on average. However, the trip frequency trends are quite different with no discernible difference between groups.

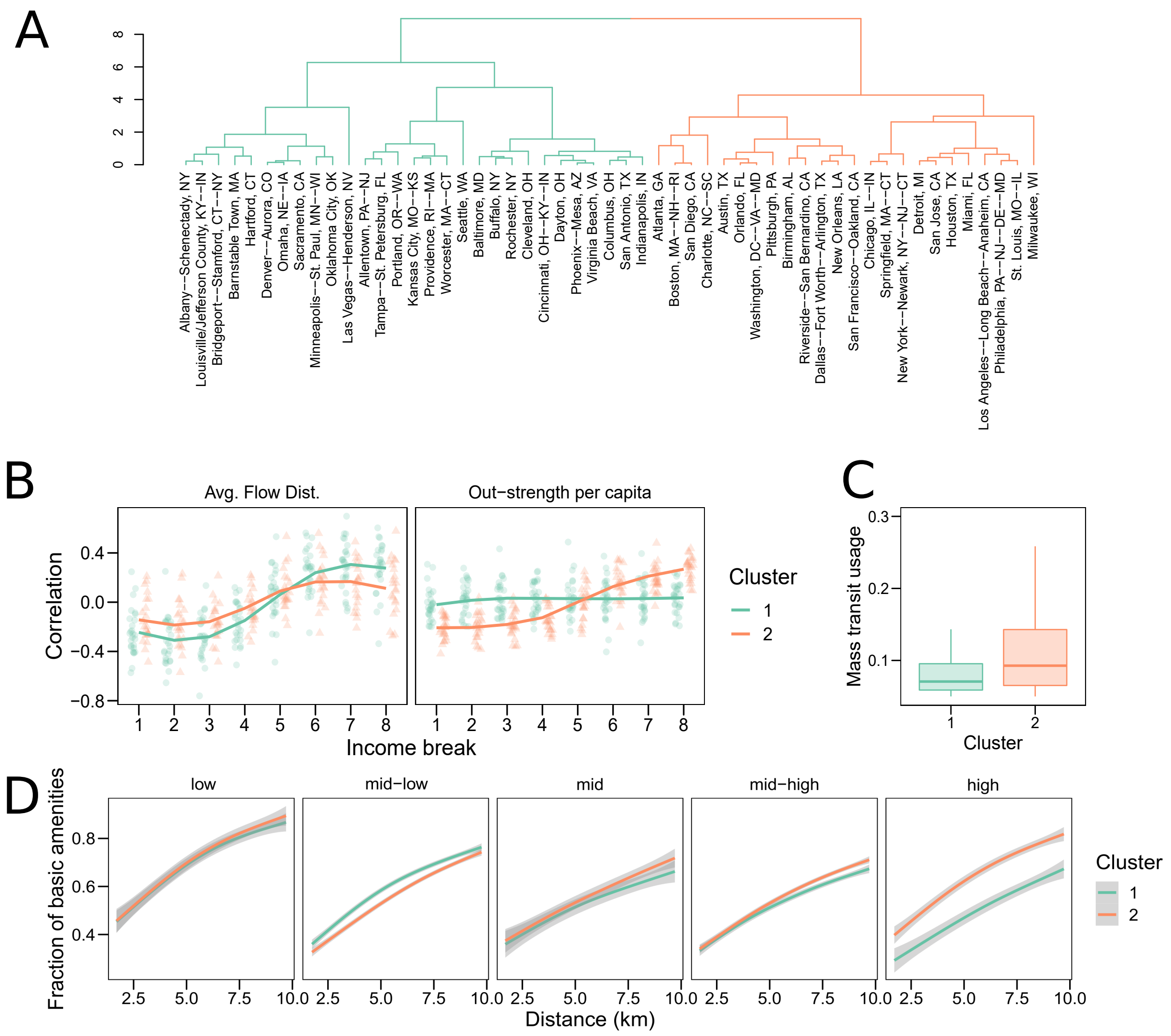

Figure S7: Reproduction of core findings using LODES commuting dataset. **A** The identified city clusters are similar (Fowlkes-Mallows score = 0.8), though not identical, to the clusters shown in Fig. 4. **B** The trends in the correlation between income and average flow distance are similar to, but less exaggerated than, those found when using location data. The correlation with out-strength per capita however is quite different. **C** The difference in mass transit usage between clusters **D** Finally while we see limited differences in amenity distance between clusters at most income levels, we find that in the teal cluster, high-income individuals live on average further from basic amenities, similar to the trend seen in Fig. 4.

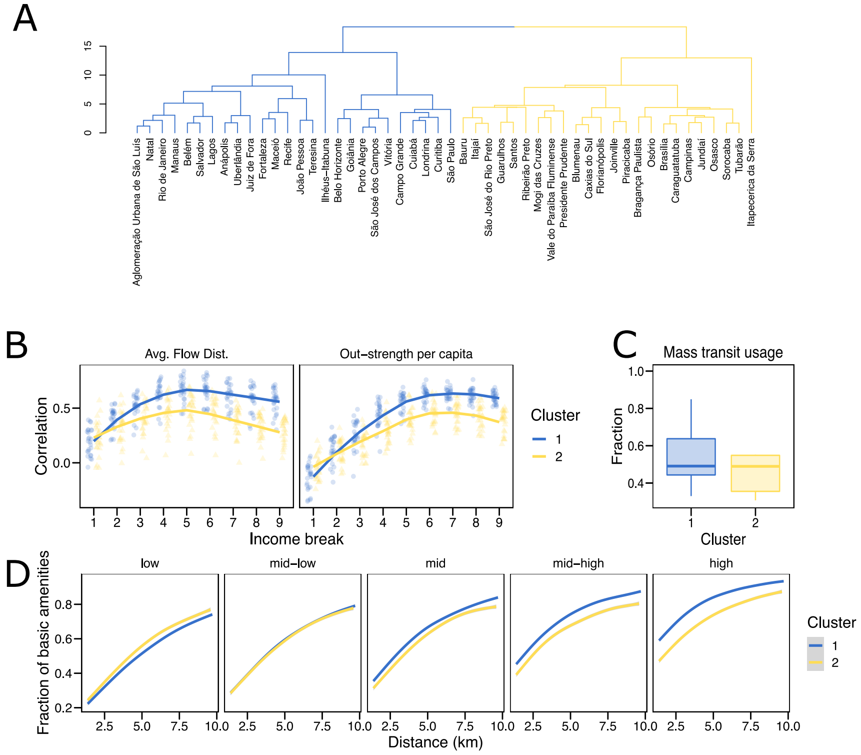

Figure S8: **A.** Dendrogram obtained from socio-mobility clustering analysis in Brazilian cities. Cluster 1 (blue) contains almost all the largest metropolitan areas and the capital cities of their respective states, with the exceptions of Lagos and São José dos Campos whereas among those in Cluster 2 (yellow) the only state capital is Florianópolis. **B** Spearman correlation between the number residents of each income bracket and the average flow distance and out-strength per capita. All cities show a high correlation, and the only difference between them is the magnitude of such correlation and how it affects the middle class. **C** Fraction of mass transit usage for each of the clusters. **D** Fraction of basic amenities as a function of distance for 5 income levels.

S6 Amenity diversity

The amenity diversity was defined as the Shannon entropy, computed from the relative frequencies of the amenities' distribution. In our analyses we considered a set eight major categories of amenities, namely: $A = \{$`art-culture,education,entertainment, finance, healthcare, sustenance, transportation, other`$\}$. For each spatial location unit l we computed the entropy

$$H_l^a = -\sum_{i \in l} p_i \log p_i$$

where p_i is the fraction of amenities in the area such that $p_i = \frac{n_i}{N}$ with N being the total number of amenities in the area and n_i the number of amenities of type i.

S7 Spatial autocorrelation of areas according to income

The spatial autocorrelation of median incomes across zip codes, is calculated as:

$$I = \frac{N}{W} \frac{\sum_{i,j} w_{ij}(x_i - \bar{x})(x_j - \bar{x})}{\sum_i (x_i - \bar{x})^2} \tag{S1}$$

where N is the total number of zip codes i in a city, x_i is the median income of zip code i, $\bar{x}$ is the mean of all median incomes in the city, $w_{ij} = 1$ if zip code j is touching i and 0 otherwise, and W is the sum of all w_{ij}.

The metric measures how alike zip codes are to their direct neighbors within a city by comparing them to the city-average. I is near -1 for a totally mixed distribution of zip code incomes (zip codes are unlike their neighbors relative to the average, leading to negative numerator contributions), 0 for a random arrangement, and 1 if high and low income zip-codes are completely separated (zip codes are completely alike their neighbors, leading to positive numerator contributions.) We can interpret the autocorrelation of median income as a measure of the degree to which incomes are spatially mixed within a city. Higher values indicate that zip codes of a particular income tend to be grouped together (as in the presence of "downtown areas",) whereas lower values indicate a more homogeneous distribution throughout a city.
